# Thyroid function in adult Nigerians with metabolic syndrome

**DOI:** 10.11604/pamj.2014.18.352.4551

**Published:** 2014-08-29

**Authors:** Ifeoma Udenze, Ilochi Nnaji, Temitope Oshodi

**Affiliations:** 1 Department of Clinical Pathology, Faculty of Clinical Sciences, College of Medicine, University of Lagos, Lagos, Nigeria

**Keywords:** Thyroid, diastolic blood pressure, euthyroid syndrome

## Abstract

**Introduction:**

Metabolic syndrome and thyroid dysfunction are two common disorders encountered in the metabolic clinic. Recently, there has been increased interest in the association between the two disorders because of the similarities between symptoms of hypothyroidism and components of the metabolic syndrome. While some reports suggest that metabolic syndrome is associated with subclinical hypothyroidism, this concept is largely under investigated in Nigerian adults with metabolic syndrome. The aim of this study is to determine the thyroid function status of adult Nigerians with metabolic syndrome and determine the association, if any, between metabolic syndrome and thyroid function.

**Methods:**

This was a cross sectional study of one hundred and fifty adults, members of staff of the College of Medicine of the University of Lagos. The participants were recruited using a cluster random sampling method. The Ethical Research & Review Committee of the institution approved the study protocol and signed informed consent was obtained from the participants. The statistics was analysed using the IBM SPSS Software of version 19.0. The Student's t test, Chi square test and multivariate regression analysis were employed for the analysis. Statistical significance was set at p < 0.05.

**Results:**

Thirty nine (twenty-six percent) of the study participants had metabolic syndrome and one hundred and eleven (seventy-four percent) of the study participants did not have metabolic syndrome, served as controls. Those who had metabolic syndrome group were significantly older (p = 0.03), metabolic syndrome was significantly associated with the female gender (p = 0.0002), higher systolic blood pressure (p = 0.0034), diastolic blood pressure (p = 0.0009), waist circumference (p < 0.0001), body mass index (p < 0.0001), waist-hip ratio (p = 0.003), fasting serum glucose (p = 0.0457) and free thyroxine (fT4) levels (p = 0.0496). Those with metabolic syndrome had significantly lower HDL (P = 0.004) and free triiodothyronine (fT3) levels (p = 0.037). There was no statistically significant difference in the thyroid stimulating hormone (TSH) levels between individuals with and without metabolic syndrome. Thirty-three percent of the metabolic syndrome cases had sick euthyroid syndrome (p= < 0.0001). In multivariate regression, waist circumference was significantly and inversely associated with the sick euthyroid syndrome (p = 0.011).

**Conclusion:**

Metabolic syndrome is associated with the sick euthyroid syndrome in adult Nigerians. Abdominal obesity appears to be the link between metabolic syndrome and the sick euthyroid syndrome.

## Introduction

The metabolic syndrome is associated with increased life-time risk for atherosclerotic cardiovascular disease [1]. The prevalence of metabolic syndrome has increased greatly not only in industrialized nations [2] but in developing countries as well [3, 4]. Metabolic syndrome and thyroid dysfunction are encountered commonly in the metabolic and endocrinology clinics, often times occurring together [5, 6]. Some studies have published a high prevalence of thyroid disorders in individuals with metabolic syndrome [5, 6]. Recently there has been increased interest in the association between thyroid function and metabolic syndrome based on the notion that triiodothyronine controls metabolic and energy homeostasis and influences body weight, thermogenesis, lipolysis and metabolism of cholesterol [7].

The criteria defining metabolic syndrome according to the National Cholesterol Education Programme-Adult Treatment Panel III (NCEP-ATPIII) [8], require a combination of at least 3 of the following 5 criteria: Abdominal circumference ≥ 102 cm in males or ≥ 88 cm in females, HDL cholesterol < 1.03 mmol/L (< 40 mg/dL) (males) or < 1.3 mmol/L (< 50 mg/dL) (females), triglycerides ≥ 1.7 mmol/L (≥ 150 mg/dL), blood pressure ≥ 130/85 mmHg or the patient receiving hypotensive treatment and fasting glycaemia > 6.1 mmol/L (> 110 mg/dL). This cluster of metabolic abnormalities is associated with increased risk for atherosclerotic cardiovascular disease and type 2 diabetes mellitus [9].

In hypothyroidism, there is increase in low density lipoprotein cholesterol (LDL), total cholesterol (TC) triglyceride (TG) and weight gain which may lead to obesity, and an increase in blood pressure [10]. These similarities in symptoms of obesity, dyslipidaemia and dysglycemia in both disorders have lead researchers to posit that metabolic syndrome may be a consequence of some occult abnormality of the thyroid gland [11, 12]. Other studies have also pointed to thyroid disorders as being complications of the metabolic syndrome and type 2 diabetes [13, 14]. Many researchers have documented an association between components of the metabolic syndrome and serum levels of TSH, fT4 and fT3 [15–18]. Many studies have associated subclinical hypothyroidism with metabolic syndrome [5, 6, 11]. Other studies have found the same associations in the older age group [19, 20]. There is paucity of data on thyroid function in metabolic syndrome in adult Nigerians. The aim of this study therefore is to determine the association of thyroid disorder with metabolic syndrome in adult Nigerians.

## Methods

### Study Design

This was a cross sectional study of one hundred and fifty adults, males and females, members of staff of the College of Medicine of the University of Lagos. The participants were recruited using a cluster random sampling method. The units in the university were grouped into clusters. The cluster to be studied was selected by simple random sampling method. Every individual within the cluster who met the selection criteria, and who gave consent, was recruited for the study. The Ethical Research & Review Committee of the institution approved the study protocol and informed consent was signed and obtained from the participants.

### Inclusion and Exclusion Criteria

The inclusion criterion was adult males and females between 35 and 70 years of age. Adults with overt thyroid disease, on steroid medication and known diabetics were excluded from the study. Pregnant women were also excluded from the study. Individuals who met the above criteria and who agreed to participate in the study, reported on the morning of the study after an overnight (10-12 hours) fast. 5mls of venous blood was collected from the ante cubital vein. Anthropometric measurements were taken. Abdominal obesity was determined by measurement of the waist circumference in centimetres, using the pubic crests and the umbilicus as land marks. The hip circumference was measured from the farthest point on the gluteus using the anatomical neck of the femur as land marks. The blood pressure was determined using the Accoson's Mercury Sphygmomanometer (cuff size 15×43cm). The subjects were seated and rested for 30minutes before measurement. The systolic blood pressure was taken at the first korotkoff sound and diastolic at the fifth korotkoff sound. The average of two readings taken fifteen minutes apart was used. The total, LDL, HDL cholesterol, triglyceride and glucose concentrations were determined on fasting serum samples [21] using reagents from Randox Laboratories Limited, Antrim, UK, BT 29 4QY, on semiautomatic biochemistry analyser BS3000P-Sinnowa Medical Science and Technology company limited, Nanjing, China (211135). Free T3, T4 and TSH concentrations were determined using reagents from Inteco Diagnostics, UK, E8 3DY, by an enzyme linked immunoassay technique [22] on Acurex Plate Read - Acurex Diagnostics, Ohio, USA (419-872-4775)

### Statistical analysis

The data was analysed using the IBM SPSS Software of version 19.0. The Student's t test was used to test the differences in the mean values for the continous variables. Chi square test was used to test the differences in proportion of the catergorical variables. Multivariate regression analysis was also employed to test for strength of relationship between continuous variables. Statistical significance was set at *p*< 0.05.

## Results

Table 1 shows the clinical and laboratory characteristics of subjects with and without metabolic syndrome. Systolic blood pressure was significantly higher in people with metabolic syndrome (p = 0.003), diastolic blood pressure was also significantly higher in the metabolic syndrome group (p = 0.0009). Waist circumference and the fasting plasma glucose levels were also significantly higher in the group with metabolic syndrome (p < 0.0001 and p = 0.045 respectively). HDL cholesterol levels were significantly lower in the metabolic syndrome group (p = 0.004). fT3 was significantly lower and fT4 levels significantly higher in the group with metabolic syndrome (p = 0.037 and 0.049 respectively. After adjustment for age and sex, these associations remained significant. Systolic blood pressure (p = 0.028), diastolic blood pressure (p = 0.0013), waist circumference (0.017), plasma glucose (p = 0.049), HDL (p = 0.034), fT3 (p = 0.042), fT4 (p = 0.039).Table 2 shows the thyroid function status of the study participants The sick euthyroid syndrome was significantly associated with metabolic syndrome while most of the controls were euthyroid.Table 3 shows the multivariate regression of components of the metabolic syndrome on sick euthyroid syndrome. Only waist circumference showed an association with the sick euthyroid syndrome.

**Table 1:** Clinical and laboratory characteristics of subjects with and without metabolic syndrome

Characteristics	Metabolic syndrome N = 39/ (%) Mean±SD	No metabolic syndrome N = 111/ (%) Mean±SD	P value
Gender			
Females	31(79.5)	50(45)	0.0002*
males	8(20.5)	61(54.9)	
Age(years)	49.61±7.84	46.33±8.10	0.030*
SBP(mmHg)	131.97±17.54	122.42±17.14	0.003*
DBP(mmHg)	83.10±11.25	75.77±11.78	0.0009*
WC(cm)	99.61±9.11	89.77±9.37	<0.0001*
BMI(kg/m^2^)	30.60±4.41	26.37±5.93	<0.0001*
WHR	0.88±0.05	0.85±0.05	0.003*
Glucose (mmoles/L)	5.24±2.07	4.61±1.54	0.045*
TG (mmoles/L)	1.90±0.13	1.84±0.20	0.091
HDL (mmoles/L)	1.26±0.12	1.33±0.14	0.004*
TC (mmoles/L)	5.02±0.44	5.16±0.42	0.08
LDL (mmoles/L)	2.90±0.42	2.99±0.45	0.26
T_3_ (mmoles/L)	2.86±1.24	3.92±3.09	0.037*
T_4_ (mmoles/L)	17.24±9.94	14.07±8.05	0.049*
TSH (µiU/ml)	1.06±0.88	1.10±0.88	0.794

* Statistically significant

**Table 2:** Thyroid function in the study participants

Thyroid state	Metabolic syndrome N = 39(%)	No metabolic syndrome. N= 111(%)	P value
Sick euthyroid syndrome	13(33.3)	2(1.8)	<0.0001*
Euthyroidism	23(58.9)	88(79.2)	<0.0001*
Primary hyperthyroidism	1(2.6)	2(1.8)	0.986
Secondary hypothyroidism	1(2.6)	4(3.6)	0.973
Subclinical hyperthyroidism	1(2.6)	5(4.5)	0.856
Subclinical hypothyroidism	0(0)	0(0)	

**Table 3:** Multivariate regression of components of the metabolic syndrome on sick euthyroid syndrome

Components of the Metabolic syndrome	Regression coefficient	P value
SBP(mmHg)	0.75	0.208
DBP(mmHg)	0.92	0.126
WC (cm)	-0.060	0.011*
Glucose (mmoles/L)	0.074	0.613
TG (mmoles/L)	-1.14	0.375
HDL (mmoles/L)	1.596	0.341

* Statistically significant

## Discussion

The study identified sick euthyroid syndrome as the commonest abnormality of the thyroid in adult Nigerians with metabolic syndrome. Low fT3 levels which is the most consistent finding in sick euthyroid syndrome was significantly associated with the metabolic syndrome and had mean values significantly lower in the metabolic syndrome. The ability of the study to bring out these findings highlights its strength. The study contributes to medical practise as awareness of these alterations will help in avoiding errors in diagnosis of thyroid disorders and inappropriate therapy. The limitations of the study include unavailability of electronic health records/poor record keeping in the communities which would enable access to data from a larger section of the population at significantly lower cost. This study showed a statistically significant difference in the components of the metabolic syndrome between the study group with metabolic syndrome and the control group, in keeping with findings in literature [2, 3] and these associations remained after adjustments for age and sex (Table 1).

From this study, the sick euthyroid syndrome is the most common abnormality of the thyroid gland in people with metabolic syndrome occurring in 33.3% of the group with metabolic syndrome compared to 1.8% in the control group (p < 0.0001). This differs from findings by some authors working in different regions and with different races. Workers in India [5, 16], Taiwan [19] and Korea [18], found a high prevalence of sub clinical hypothyroidism in people with metabolic syndrome. Heima et al [23] working in Amsterdam also found an association between higher TSH levels and metabolic syndrome in euthyroid subjects. Iodine deficiency, autoimmune thyroiditis and mutations in the TSH receptor genes are some of the hypothesis put forward to explain the association between increasing TSH, obesity and subclinical hypothyrodism in these populations [7]. Longitudinal studies are required to determine whether the metabolic alterations are a cause or a consequence of the thyroid dysfunction. In these populations, a high prevalence of metabolic syndrome was also found in patients with subclinical hypothyroidism [11]. In subclinical hypothyroidism, there is altered thyroid function with normal feedback regulation (fT4 at the lower limit of normal range and increased TSH also within normal range) was thought to be the primary event that induces alterations in energy expenditure with subsequent increases in BMI and weight and other cardio metabolic risk factors [24, 25]. Studies have also recorded high prevalence rates for both subclinical hypothyroidism [26] and metabolic syndrome [5] in these regions and these may further explain the associations.

The differences in thyroid function in metabolic syndrome observed in this study may also be explained by genetic differences across regions as studies on the genetic causes of the metabolic syndrome have demonstrated no single locus reproducibly linked with the metabolic syndrome across populations, partly explained by the effect of ethinicity and by the complexity of the metabolic syndrome itself [27]. Ethnic and regional differences in reference values for TSH which appear to be lower in the Nigerian population compared to India, Europe and USA [26, 28], may also contribute to the differences in thyroid function in the metabolic syndrome. In the present study, fT3 was significantly lower and fT4 significantly higher in the group with metabolic syndrome with no significant difference in the TSH values in both groups (Table 1), this is in keeping with the sick euthyroid syndrome. In the sick euthyroid syndrome, the most consistent finding is a low or low normal fT3 value with raised reverse T3. Also seen, is a low normal or normal TSH and fT4 is normal or mildly elevated [10].

The sick euthyroid syndrome is an abnormality of thyroid hormone concentration seen in a wide variety of Non thyroidal illnesses. The pathogenesis of the disorder is associated with inhibition of the hepatic enzyme, 51 monodeiodinases which catalyzes the conversion of fT4 to active fT3 with increase in reverse T3 [10]. These changes may also be mediated in part by inflammatory cytokines acting at the level of the hypothalamus and pituitary [29]. It is not clear whether it is an adaptive response which lowers tissue energy requirement during systemic illness or a maladaptive response leading to damaging tissue hypothyroidism [29]. The thyroid abnormalities normalize as the patient recovers from illness [10]. Treatment of obesity with hypocaloric diet also causes changes in thyroid function resembling the sick euthyroid syndrome [30], although the effect of diet therapy was not assessed in the present study. Longitudinal studies looking at the long term effects of diet therapy and long term effects of low fT3 values in these individuals are needed. It is possible that this tissue hypothyroidism could set off a sequence of metabolic dysregulations to worsen the individual's metabolic condition.

In this study, employing multivariate regression, central obesity determined by the waist circumference was associated with the sick euthyroid syndrome (Table 3). Some studies also found associations between thyroid dysfunction and obesity in the metabolic syndrome [7, 31]. Obesity appears to be the link between metabolic syndrome and the sick euthyroid syndrome. Fat cells produce leptin and are thus considered an active endocrine organ [24]. In addition to the role of leptin in regulating energy homeostasis, [24] leptin is also an important neuroendocrine regulator of the hypothalamic-pituitary-thyroid axis [32] by regulation of TRH gene expression in the paraventricular nucleus. Leptin also affects thyroid deiodinase activities with activation of T4 to T3 conversion [24, 33]. Available data support the concept of an inverse relationship between thyroid hormone and leptin [24, 32, 33] and would explain the inverse relationship between waist circumference and sick euthyroid syndrome found in this study (Table 3).

## Conclusion

Metabolic syndrome is associated with changes in thyroid function, in adult Nigerians, consistent with the sick euthyroid syndrome and obesity appears to be the link between metabolic syndrome and the sick euthyroid syndrome. At present, the patients with sick euthyroid syndrome are considered to be essentially euthyroid and thyroid replacement therapy is not administered. Awareness of these alterations helps in avoiding errors in diagnosis of thyroid disorders and inappropriate therapy. Further studies are however needed to examine the long term effects of low fT3 on the metabolic status of individuals with metabolic syndrome.
